# A symptom-related monitoring program following pulmonary embolism for the early detection of CTEPH: a prospective observational registry study

**DOI:** 10.1186/1471-2466-14-141

**Published:** 2014-08-28

**Authors:** Matthias Held, Alexander Hesse, Franziska Gött, Regina Holl, Gudrun Hübner, Philipp Kolb, Heinz Jakob Langen, Tobias Romen, Franziska Walter, Hans Joachim Schäfers, Heinrike Wilkens, Berthold Jany

**Affiliations:** 1 Medical Mission Hospital, Department of Internal Medicine, Academic Teaching Hospital of the Julius-Maximilian University of Würzburg, Salvatorstrasse 7, Würzburg 97067, Germany; 2 Firestone Institute for Respiratory Health, Department of Medicine, Pathology & Molecular Medicine, McMaster University, 50 Charlton Ave East, T2131, Hamilton, ON L8N 4A6, Canada; 3 Medical Mission Hospital, Department of Radiology, Academic Teaching Hospital of the Julius-Maximilian University of Würzburg, Salvatorstrasse 7, Würzburg 97067, Germany; 4 University Hospital Homburg Saar, Clinic for Thoracic and Cardiovascular Surgery, Kirrberger Strasse, Homburg, Saar D 66421, Germany; 5 Department of Internal Medicine V, Respiratory and Critical Care Medicine, University Hospital Homburg Saar, Kirrberger Strasse, Homburg, Saar D 66421, Germany

**Keywords:** Dyspnea, Cardiopulmonary exercise testing, Pulmonary artery, Pulmonary artery pressure, Chronic thromboembolic pulmonary hypertension, Follow-up, Pulmonary embolism, Pulmonary hypertension, Pulmonary circulation

## Abstract

**Background:**

Chronic thromboembolic pulmonary hypertension (CTEPH) is a long-term complication following an acute pulmonary embolism (PE). It is frequently diagnosed at advanced stages which is concerning as delayed treatment has important implications for favourable clinical outcome. Performing a follow-up examination of patients diagnosed with acute PE regardless of persisting symptoms and using all available technical procedures would be both cost-intensive and possibly ineffective. Focusing diagnostic procedures therefore on only symptomatic patients may be a practical approach for detecting relevant CTEPH.

This study aimed to evaluate if a follow-up program for patients with acute PE based on telephone monitoring of symptoms and further examination of only symptomatic patients could detect CTEPH. In addition, we investigated the role of cardiopulmonary exercise testing (CPET) as a diagnostic tool.

**Methods:**

In a prospective cohort study all consecutive patients with newly diagnosed PE (n=170, 76 males, 94 females within 26 months) were recruited according to the inclusion and exclusion criteria. Patients were contacted via telephone and asked to answer standardized questions relating to symptoms. At the time of the final analysis 130 patients had been contacted. Symptomatic patients underwent a structured evaluation with echocardiography, CPET and complete work-up for CTEPH.

**Results:**

37.7%, 25.5% and 29.3% of the patients reported symptoms after three, six, and twelve months respectively. Subsequent clinical evaluation of these symptomatic patients saw 20.4%, 11.5% and 18.8% of patients at the respective three, six and twelve months time points having an echocardiography suggesting pulmonary hypertension (PH). CTEPH with pathological imaging and a mean pulmonary artery pressure (mPAP) ≥ 25 mm Hg at rest was confirmed in eight subjects. Three subjects with mismatch perfusion defects showed an exercise induced increase of PAP without increasing pulmonary artery occlusion pressure (PAOP). Two subjects with pulmonary hypertension at rest and one with an exercise induced increase of mPAP with normal PAOP showed perfusion defects without echocardiographic signs of PH but a suspicious CPET.

**Conclusion:**

A follow-up program based on telephone monitoring of symptoms and further structured evaluation of symptomatic subjects can detect patients with CTEPH. CPET may serve as a complementary diagnostic tool.

## Background

The incidence of chronic thromboembolic pulmonary hypertension (CTEPH) after an acute pulmonary embolism (PE) varies between 0.5 – 8% depending on the study population [1-4]. Inclusion of patients with unprovoked PE [4] and subjects with a history of previous pulmonary embolism results in a cohort with a higher incidence of CTEPH [5]. Exclusion of patients with comorbidities could lead to a lower incidence rate. There is still an ongoing debate about the true incidence of CTEPH. It is critical to note that patients are frequently diagnosed with CTEPH at advanced stages of the disease leading to worse clinical outcomes which could theoretically be alleviated through earlier treatment [6,7]. Registry data show that the majority of patients undergoing pulmonary thrombendarterectomy (PEA) are at WHO functional class III [8]. Recent data suggests that on average an 18 to 24 months delay exists from onset of symptoms to the final diagnosis of CTEPH [9]. Although modern management of CTEPH including PEA in operable patients and the use of targeted therapies improved survival [6,8], CTEPH is still a disease with a serious prognosis [6]. Since outcome remains poor in non-treated patients [10,11] and is dependent of WHO functional class [7,8], late detection of CTEPH might lead to an even worse prognosis. Among patients who underwent PEA in-hospital survival as well as 1-year-survival was associated with the time elapsed between the last pulmonary embolism and PEA [8]. Recent data suggests that patients with perfusion abnormalities and a borderline PH at rest, but an increase under exercise without an increase of pulmonary arterial occlusion pressure improve following an early pulmonary thrombendarterctomy [9]. Overall, evidence from various sources suggests that early detection and treatment of CTEPH is advantageous in achieving favourable clinical outcomes.

Incomplete thrombus resolution after pulmonary embolism is not a rare finding [12]. Given the incidence of pulmonary embolism of 0.6 -1.45/1000 person-years [13-15], a high rate of undiagnosed CTEPH is very likely. Symptoms of pulmonary hypertension are unspecific [16]. This might contribute to a still existing delay from onset of symptoms to diagnosis of CTEPH [9], especially in elderly patients.

Although Echocardiography is currently used as a method to detect elevated systolic right ventricular pressure, it has been associated with false negative results and may not always detect pulmonary hypertension. [17-22]. Cardiopulmonary exercise testing can help to distinguish CTEPH from PAH [23] and has been suggested as a reliable method to detect CTEPH when echocardiography results are negative [17]. This suggests that it could be a helpful tool for early diagnosis of CTEPH.

Incomplete thrombus resolution after acute pulmonary embolism without clinical symptoms has been reported [24,25]. It would be interesting to see quantitative and qualitative data on asymptomatic subjects with pathological VQ-scan and incomplete thrombus resolution developing CTEPH. However, complete follow-up examination using all technical procedures available in all patients at the first step, such as echocardiography, CT-scan, V/Q scan of all patients with acute PE could be cost-intensive and ineffective in detecting CTEPH. Focusing further structured diagnostic evaluation on symptomatic patients only may therefore be an ethical and effective approach and a concept in detecting relevant CTEPH.

### Objective

As there are currently no established structured follow-up programs for early detection of CTEPH, this study aimed to evaluate a novel follow-up program based on a telephone monitoring of patient reported symptoms and consecutive step by step evaluation for patients with pulmonary embolism to detect CTEPH. Furthermore this study evaluated cardiopulmonary exercise testing (CPET) as a complementary diagnostic tool for early detection of CTEPH.

## Methods

**Figure 1:**
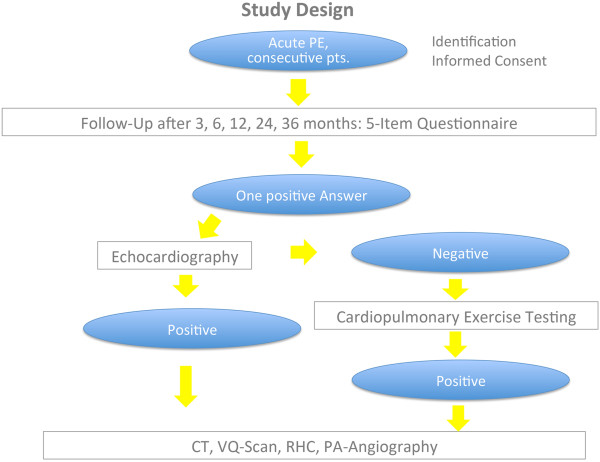
Study design.

The study design is summarized in Figure 1. In an ongoing observational program, we prospectively studied all consecutive patients, who presented with newly diagnosed PE at the Medical Mission Hospital from January 2011 to February 2013. We did not exclude any patients with comorbidities or a history of previous pulmonary embolism. After written informed consent was obtained we included patients with idiopathic as well as patients with provoked pulmonary embolism. Patients at least 18 years old were included and were contacted after three, six, twelve, 24 and 36 months by a standardized telephone call in which they were interviewed according to a questionnaire for the following symptoms: dyspnea at rest, dyspnea on exertion, dizziness, fainting or syncope or thoracic pain. The online Additional files 1 and 2: Figure S1-S2 detail this questionnaire. If any item of a five item-questionnaire was reported as positive patients were invited for an outpatient visit including an echocardiography. Echocardiography (Vivid7®, GE Medical Systems, Solingen, Germany) was performed according to the guidelines [19,26-28]. If the echocardiography suggested normal results, a CPET was performed. An echocardiography or a CPET indicative of PH warranted further evaluation.

CPET (Masterscreen CPX®, CareFusion, Hoechberg, Germany; E-bike basic PC plus, GE Medical Systems, Solingen, Germany) was performed according to the ATS and American College of Chest Physicians (ACCP) Statement as recently described including a two-minute registration at rest and two-minute recording of unloaded pedaling [29,30]. The following exercise protocol consisted of an increasing work load of 25 Watt /2 min per ramp. We terminated exercise by symptom limitation or when withdrawal criteria were met. Respiratory rate and minute ventilation as well as expiratory fraction of O_2_ and CO_2_ were measured breath by breath. Temperature and air pressure were recorded continuously. Anaerobic threshold was determined at EQO_2_ nadir. The following parameters were assumed as indicative for disturbed pulmonary perfusion and leading to a recommendation for a complete diagnostic work-up: EQO_2_ > 25, EQ CO_2_ > 35, VE/VCO_2_ > 35, PET CO_2_ < 35 mmHg or decreasing during exercise, PaETCO_2_ > 0 mmHg at maximum load, and/or P(A-a)O_2_ > 35 mm Hg at the peak exercise.

Further evaluation was conducted as described above if either the echocardiography showed signs of pulmonary hypertension, or if echocardiography was normal and CPET was suggestive of pulmonary perfusion abnormalities [17].

A complete diagnostic work-up according to the current guidelines including imaging studies and right heart catheterization was suggested as a procedure agreeing with the standard care. CTEPH was assumed when mismatched perfusion defects were detected by VQ scan (Technegas-Generator®, Tetley Medical Limited, Australia; E Cam Variable®, Siemens Medical Solutions Inc., Hoffman Estates, Illinois, USA) or computed tomography scan (Activion 16®, Toshiba Medical Systems, Neuss, Germany) showed filling defects and right heart catheter revealed precapillary pulmonary hypertension [26,27]. Right heart catheterization was performed according to the guidelines using a Swan Ganz catheter (Smith Medical, Grasbrunn, Germany) [26,31]. Measurements were conducted with the monitor system IntelliVue MP70 (M8007A)®, Philips Medizinsysteme, Böblingen, Germany. For confirmation of CTEPH and for the operability assessment a pulmonary angiography (Integris Allura; Philips Medical Systems, Best, The Netherlands, films stored digitally) was performed as part of standard care.

Operability was then evaluated by a multidisciplinary team consisting of a respiratory physician, cardiologist, radiologist and a surgeon specialized and highly experienced in pulmonary thrombendarterectomy.

Written informed consent was obtained from all patients prior to inclusion. The study was approved by the local Ethics Committee of the Julius Maximilian University of Würzburg and was performed according to the Declaration of Helsinki.

## Results

As this study is still recruiting patients and undergoing an extensive follow-up program for patients with acute pulmonary embolism the data collection is not yet finalized.

26 months after this program has started 130 patients had passed a time point of three-months-follow-up, 102 patients had completed six-months follow-up and 58 patients had passed the point of twelve-months-follow up. Table 1 shows the anthropometric baseline data of the 130 patients who had passed the 3-months follow-up period. Table 2 shows the results of the telephone calls and clinical data after visits at three, six and twelve months.

**Table 1:** **Anthropometric data and comorbidities at baseline of the 130 patients who had been contacted at 3 months of follow**-**up**, **data is from the analysis on February 28 **^**th **^**2013**

**Baseline data**	**n**	**%**
Patients contacted	130	
Sex m/f	55/75	42.3%/57.7%
	Mean	+/-SD
Age (years)	65.7	17.0
Height (cm)	170	9.5
Weight (kg)	82.5	17.2
BMI (kg/m^2^)	28.6	5.4
Comorbidities	n	%
Arterial hypertension	55	42.3
Coronary artery disease	11	8.5
Atrial fibrillation/flutter	19	14.6
Heart valve pathology	8	6.2
COPD	10	7.7
Asthma	8	6.2
Interstitial lung disease	6	4.6
Sleep related breathing disorder	11	8.5
Alveolar hypoventilation	1	0.8
Diabetes	15	11.5
Chronic kidney disease	9	6.9
Liver disease	6	4.6
Thyroid disorder/Struma	25	19.2
Rheumatism/collagen vascular disease	7	5.4

**Table 2:** **Results of telephone monitoring and administration of 5**-**item**-**questionnaire**, **echocardiography**, **cardiopulmonary exercise testing and further evaluation confirming CTEPH**

	**Visit 1, 3 months**	**Visit 2, 6 months**	**Visit 3, 12 months**
	**N(%)**	**N(%)**	**N(%)**
Telephone calls	130	102	58
5-Item-Questionnaire positiv	49 (37.7%)	26 (25.5%)	17 (29,3%)
Outpatient visits	49	26	17
Echocardiography,	n = 49	n = 26	n = 16
RVSP >/=35 mm Hg	10 (20.4%)	3 (11.5%)	3 (18.8%)
REVSP < 35 mm Hg/ not detectable	39 (79.6%)	23 (88.5%)	13 (81.25%)
Cardiopulmonary exercise testing,	n = 34	n = 15	n = 9
Abnormal pulmonary perfusion suspected	12 (35.28%)	4 (26.7%)	1 (11.1%)
Further evaluation	21	7	4
CTEPH/CTPVD proven per visit	5(3.85)/2(1.5%)	3(2.94/1(0.98%)	0/0.0%)
CTEPH/CTPVD proven, all patients	5(3.85%)/2(1.5%)	8(6.2%)/3(2.3%)	8(6.2%)/3(2.3%)

26 months after the start of the program 130 patients had passed 3-months follow-up, 102 patients had completed 6 months follow-up and 58 patients had passed twelve months-follow-up.

Three months post diagnosis 49 patients (37.7%) out of 130 patients who had completed the follow-up period and had been contacted and interviewed via telephone reported at least one symptom out of five outlined in the questionnaire. In all 49 symptomatic patients an echocardiography was performed. Echocardiography showed a right ventricular systolic pressure (RVSP) of at least 35 mmHg in ten (20.4%) patients suggesting a positive readout for PH. 39 patients (79.6%) saw negative results from the echocardiography. 34 of these 39 subjects underwent subsequent CPET. Of these 34 patients, 12 (35%) patients had CPET results suggesting CTEPH. In total 21 patients of the 22 who had test results suggesting CTEPH underwent a complete work-up for CTEPH. In one patient with a VQ-scan suggesting CTEPH this diagnosis was ruled out by pulmonary angiography. In five subjects CTEPH was confirmed. In two subjects chronic thromboembolic pulmonary vascular disease (CTPVD) with an exercise induced PH (normal pulmonary artery pressure at rest, but an exercise-induced increase of mean pulmonary artery pressure (mPAP) without an increase of pulmonary artery wedge pressure (PAWP) was confirmed.

In four subjects with suspicion of PH a complete work-up was recommended, but right heart catheterization was delayed by the patients. Comorbidities of the 49 subjects with reported symptoms were arterial hypertension (14/49), malignancies (8/49), coronary artery disease (6/49), chronic obstructive pulmonary disease (5/49), atrial fibrillation (3/49), asthma (3/49), interstitial lung disease (3/49) and cardiac valve pathology (1/49).

After six months 26 out of 102 (25.5%) patients who had passed the six-month follow-up period were symptomatic and were invited in an outpatient visit as described. RVSP was at least 35 mmHg in three patients (11.5%) and normal in 23 patients (88.5%). Abnormal pulmonary perfusion was suspected by CPET in 4 out of 15 (26.7%) patients. Following echocardiography testing and CPET seven patients underwent a complete clinical evaluation in which CTEPH was diagnosed in three patients and CTPVD in one patient. These four patients were symptomatic at the three months visit and a complete work-up had been recommended at this time.

After twelve months 17 out of 58 (29.3%) patients who had passed the 12-months-follow-up period reported symptoms and were invited to participate in an outpatient visit as described. Echocardiography was performed in 16 subjects and revealed an RVSP of at least 35 mm Hg in three patients (18.8%). Echocardiography was normal in 13 patients (81.2%). Of these subjects one showed a CPET suggestive of a disturbed pulmonary perfusion. However, CTEPH was not confirmed in any further patient at visit 3 after 12 months.

Taken together, after the 26 months of interim analysis, out of 130, 104 and 58 patients who had passed the three, six- and twelve month follow-up period respectively, in total eight patients were diagnosed with CTEPH defined by a mean pulmonary artery pressure at rest of ≥ 25 mm Hg and pathological imaging. One of these patients had a pulmonary artery occlusion pressure suggestive for additional diastolic left ventricular dysfunction. Three additional subjects with mismatched perfusion defects detected by VQ scan showed an mPAP < 25 mm Hg at rest with an increase of mPAP while exercising without an increasing pulmonary artery occlusion pressure.

Two subjects with precapilary pulmonary hypertension at rest and one with an exercise induced increase of mPAP with normal pulmonary arterial occlusion pressure showed a pathological VQ scan without echocardiographic signs of pulmonary hypertension but a cardiopulmonary exercise test consistent with PH.

26 months after the start of the program 15 of the 130 contacted patients had died. There were four additional drop-outs described in Table 3. Table 4 shows the hemodynamic data of the eight patients with CTEPH and 3 subjects with CTPVD. Statistics are described as Mean ± SD. One patient presented with severe PH and two patients showed moderate PH. The other subjects had a mildly elevated pulmonary artery pressure at rest.

**Table 3:** **Results from the analysis 27 months after the start of the follow**-**up program**: **deaths**, **drop**-**outs and cases with confirmed CTEPH are shown in relation to the subjetcs who passed three**-**months follow**-**up**

**Results: 27 months analysis**	**N**	**%**
Patients died	15	11.5%
Dropouts	4	3.0%
CTEPH/CTPVD with exercise PH*	8/3	6.2%/2.3%
CTEPH/CTPVD: “CPET pos and Echo neg“#	2/1	1.5/0.77%

*In 8 patients pathological imaging and mean pulmonary artery pressure (mPAP) at rest of ≥ 25 mmHg had been found. In 3patients pathological imaging, mPAP at rest of < 25 mmHg, but increasing mPAP under exercise without increasing pulmonary artery occlusion pressure had been found suggesting CTPVD with exercise induced PH. # 2 out of 8 (25%) patients diagnosed with CTEPH and 1 out of 3 (33%) patients diagnosed with CTPVD and exercise induced PH showed normal echocardiography, but findings in the cardiopulmonary exercise test suspicious for functional relevant pulmonary perfusion abnormalities.

**Table 4:** Haemodynamic data of eleven patients with pathological imaging findings

	**mPAP at rest >/=25 mm Hg (n = 8)**		**mPAP at rest < 25 mm Hg (n = 3)**	
	**Mean**	**SD**	**Mean**	**SD**
mPAP (mmHg)	36	11	21	2
PAWP (mmHg)	10	3.8	9	3.6
PVR (dyn x sec x cm^-5^)	512	339	196	55
CO (l/min)*	4.5	1.2	4.77	1.0
CI (l/min/m^2^)	2.3	0.5	2.4	0.3
RAP (mmHg)	9	3	2.3	1.2
mPAP under exercise (mmHg)	-		51	8
PAWP under exercise (mmHg)	-		11	3.6

Out of eleven subjects with CTEPH or CTEPV one was not suitable for surgery due to peripheral localization of the thromboembolic lesions. For three of the remaining ten subjects surgery was not recommended after careful consideration of the potential benefit risk ratio taking into account age, comorbidities and relatively slight hemaodynamic impairment. Overall seven patients were suitable for a pulmonary thrombendarterectomy.

## Discussion

To the best of our knowledge there is no established telephone based monitoring follow-up program for CTEPH following PE. Here we show that a symptom-related telephone-monitoring based approach with consecutive diagnostic work-up of symptomatic subjects can detect CTEPH in patients after pulmonary embolism.

PE patients were included in the study regardless of comorbidities such as malignancies or a possible history of previous PE. This leads to a cohort with a CTEPH incidence of 6.2% and of 2.3% of patients with exercise induced PH and pathological imaging findings respectively. Previously, the latter condition was described as chronic thromboembolic pulmonary vascular disease [9,17]. The CTEPH detection rate is slightly higher than reported in other studies [1,3,4]. We cannot exclude that some of the subjects with detected CTEPH had an acute on chronic PE at the time of inclusion. The study, however, was not designed to find the true incidence of CTEPH, but rather aimed at providing a practical approach for detecting CTEPH early after pulmonary embolism.

The drop-out rate of this study to date has been low and a mortality rate of 11.5% was observed during follow-up. This value is not any higher than what was expected and is comparable to previous reports on cohorts of patients after acute pulmonary embolism [3,4].

The high percentage of symptomatic patients at three, six and twelve month’s follow-up is likely due to the fact that symptoms of CTEPH such as dyspnea and thoracic pain are not specific. For instance especially in an older population, left heart disease and COPD can also lead to dyspnea. The comorbidities reported in our study included airway diseases as well as arterial hypertension, coronary artery disease and atrial fibrillation. Together, these comorbidities represent the typical of a cohort of patients with the presented age and inclusion criteria.

The data acquired in this study suggests that telephone-monitoring can detect symptoms and subsequent examination of symptomatic subjects can be used to facilitate an early diagnosis of CTEPH. The questionnaire based approach however remains unspecific and does not substitute the skills of an experienced physician. As only symptomatic patients were clinically evaluated for CTEPH, we cannot exclude missing the diagnosis CTEPH in an individual asymptomatic patient. Overall, the CTEPH rate is slightly higher than that reported in research from other groups [3].

Focusing only on symptomatic patients seems to be a pragmatic approach for detecting CTEPH. This is because a follow-up program using all available diagnostic tools and techniques for detection of CTEPH after PE would be expensive and may not be cost-effective as persisting perfusion defects due to incomplete thrombus resolution without hemodynamical abnormalities has been described [24,25].

Furthermore, as two out of eight patients with diagnosed CTEPH and one out of three subjects with pathological VQ scan and exercise induced PH had a normal echocardiography but CPET pattern suggestive of CTEPH, it can be suggested that CPET may be a helpful complementary tool for the diagnosis of CTEPH. These results are in accord with recent data which also suggests CPET as a complementary tool in detecting CTEPH [17]. Another group has reported that CPET is able to differentiate CTEPH from PAH [23]. There are specific patterns leading to the suspicion of pulmonary vascular abnormalities [17].

Mean pulmonary artery pressure of the patients with confirmed CTEPH was 36 ± 11 mmHg. Three patients showed moderate to severe PH. Three patients showed an mPAP < 25 mmHg but a relevant increase of PAP under exercise conditions. In these patients PAWP was normal at rest and under exercise, so it was not considered that exercise induced PH is due to left heart diastolic dysfunction. Identification of patients with imaging findings suggesting CTEPH and normal resting hemodynamics but an increase under exercise conditions is relevant, because such patients may present as symptomatic as CTEPH patients. Drastic clinical improvement is seen in these patients following PEA [9,17].

A diagnostic and follow-up program such as the one described is intended for early diagnosis and might lead to detection of CTEPH before hemodynamics become severely disturbed.

The four subjects definitively diagnosed after six months had been symptomatic at visit one. Echocardiography and CPET led to the suspicion of PH and a complete clinical evaluation had been recommended at this time. Although our program led to the diagnosis of CTEPH after both the three and six months and did not detect further CTEPH cases at the 12-months follow-up, it cannot be concluded that a one year follow-up is sufficient for the detection of CTEPH. A “honey-moon” period after acute PE with a subsequent development of CTEPH is well described [32]. It is unclear if asymptomatic patients in this formerly reported cohort would have been detected with a structured follow-up program. This program is ongoing with planned follow-up telephone calls after two and three years in order to detect patients with slowly developing CTEPH.

Overall this registry study has several limitations: First, reported data originates from a single center. Second, due to the design of this study data from a control cohort has not been reported. It is also relevant to note that the sample size is relatively small. The questionnaire used in this study was also newly developed and therefore should be made more specific and validated towards detection of CTEPH. However, the robustness of the data as shown by right heart catheterization and imaging in all subjects following diagnosis of CTEPH and CTPVD suggests that this program could be a practical approach for diagnosis of CTEPH after acute PE.

Further advantages of a structured follow-up program could be to detect and avoid premature termination of anticoagulation therapies as well as detection of comorbidities such as cancer and cardiovascular diseases which seems to be relevant in consideration of the observed long-term mortality after acute pulmonary embolism.

## Conclusions

The symptom-related follow-up program which is based on a telephone-monitoring and a 5-item-questionnaire can be used to detect patients with CTEPH by further clinical evaluation of symptomatic patients. CPET may serve as a complementary diagnostic tool. Telephone monitoring and CPET seem to be effective and should be included in a pulmonary embolism follow-up-program used for early detection of CTEPH.

## Abbreviations

ACCP: American College of Chest Physicians; ATS: American Thoracic Society; CI: Cardiac index; CO: Cardiac output; CO_2_: Carbon dioxide; COPD: Chronic obstructive pulmonary disease; CPET: Cardiopulmonary exercise testing; CT: Computed tomography; CTEPH: Chronic thromboembolic pulmonary hypertension; EQ CO_2_: Breathing equivalent for carbon dioxide; EQ O_2_: Breathing equivalent for oxygen; mPAP: Mean pulmonary artery pressure; O_2_: Oxygen; P(A-a)O_2_: Alveolar-arterial oxygen gradient; PaETCO_2_: Arterial – endtidal carbon dioxide gradient; PAH: Pulmonary arterial hypertension; PAP: Pulmonary artery pressure; PAWP: Pulmonary arterial wedge pressure; PAOP: Pulmonary arterial occlusion pressure; PE: Pulmonary embolism; PEA: Pulmonary thrombendarterectomy; PET CO_2_: Partial pressure of endtidal CO_2_; PH: Pulmonary hypertension; SD: Standard deviation; VE/VCO_2_: Ratio of minute ventilation and carbon dioxide output; VQ scan: Ventilation/perfusion scan; WHO: World Health Organization.

## Competing interests

MH: Honoraria for lectures and/or consultancy for Actelion, Bayer Healthcare, Boehringer Ingelheim, Glaxo Smith Kline, Lilly, Novartis, Pfizer, Nycomed, Roche and Servier. AH: no conflicts of interest. FG: No conflicts of interest. R H: Honoraria for lectures, travel support and congress entry fees from Actelion, Bayer Healthcare, GSK, Novartis, OMT, Pfizer. G H: no conflicts of interest. PK: no conflicts of interests. HJL: no conflicts of interest. T R: Travel support for symposia from Glaxo Smith Kline and Heinen & Loewenstein. H J S: no conflicts of interest. FW: Travel support for symposia and conferences from Actelion, Bayer Healthcare and Glaxo Smith Kline. HW: Honoraria for consultancy, lectures and travel support for attending conferences from Actelion, Bayer, GSK, Novartis and Pfizer. B J: Honoraria for lectures from Actelion, Astra Zeneca, Boehringer Ingelheim, GSK and Novartis.

## Authors’ contributions

Conception and design: MH, AH, BJ Analysis and interpretation: MH, AH, FG, RH, GH, PK, HJL, TR, HJS, FW, HW, BJ Drafting the manuscript for important intellectual content and approving the final version: MH, AH, FG, RH, GH, PK, HJL, TR, HJS, FW, HW, BJ. All authors read and approved the final version.

## Pre-publication history

The pre-publication history for this paper can be accessed here:

http://www.biomedcentral.com/1471-2466/14/141/prepub

## Supplementary Material

Additional file 1 5 Item questionnaire, original German version. Click here for file

Additional file 2 5 Item questionnaire, translated English version. Click here for file

## References

[B1] Becattini C Agnelli G Pesavento R Silingardi M Poggio R Taliani MR Ageno W Incidence of chronic thromboembolic pulmonary hypertension after a first episode of pulmonary embolism Chest 2006 130 172 175 10.1378/chest.130.1.172 16840398

[B2] Dentali F Donadini M Gianni M Bertolini A Squizzato A Venco A Ageno W Incidence of chronic thromboembolic pulmonary hypertension in patients with previous pulmonary embolism Thromb Res 2009 124 3 256 258 10.1016/j.thromres.2009.01.003 19193397

[B3] Pengo V Lensing AW Prins MH Marchiori A Davidson BL Tiozzo F Albanese P Biasiolo A Pegoraro C Iliceto S Prandoni P theThromboembolic Pulmonary Hypertension Study Group Incidence of chronic thromboembolic pulmonary hypertension after pulmonary embolism N Engl J Med 2004 350 2257 2264 10.1056/NEJMoa032274 15163775

[B4] Klok FA van Kralingen KW van Dijk AP Heyning FH Vliegen HW Huisman MV Prospective cardiopulmonary screening program to detect chronic thromboembolic pulmonary hypertension in patients after acute pulmonary embolism Haematologica 2010 95 970 975 10.3324/haematol.2009.018960 20053871 PMC2878796

[B5] Korkmaz A Ozlu T Ozsu S Kazaz Z Bulbul Y Long-term outcomes in acute pulmonary thromboembolism: the incidence of chronic thromboembolic pulmonary hypertension and associated risc factors Clin Appl Thromb Hemost 2012 18 3 281 288 10.1177/1076029611431956 22275389

[B6] Condliffe R Kiely DG Gibbs JSR Corris PA Peacock AJ Jenkins DP Hodgkins D Goldsmith K Hughes R Sheares K Tsui SL Armstrong IJ Torpy C Cracket R Carlin CM Das C Goghlan JG Pepke-Zaba J Improved outcomes in medically and surgically treated chronic thromboembolic pulmonary hypertension Am J Respir Crit Care Med 2008 177 1122 1127 10.1164/rccm.200712-1841OC 18292468

[B7] Tscholl D Langer F Wendler O Wilkens H Georg T Schäfers H-J Pulmonary thromboendarterectomy – risk factors for early survival and hemodynamic improvement Eur J Cardiothorac Surg 2001 19 771 776 10.1016/S1010-7940(01)00686-8 11404129

[B8] Mayer E Jenkins D Lindner J D’Armini A Kloek J Meyns B Ilkjaer LB Klepetko W Delcroix M Lang I Pepke-Zaba J Simónneau G Dartevelle P Surgical management and outcome of patients with chronic thromboembolic pulmonary hypertension: results from an international prospective registry J Thorac Cardiovasc Surg 2011 141 702 710 10.1016/j.jtcvs.2010.11.024 21335128

[B9] Held M Grün M Holl R Walter F Schaefers H-J Graeter T Wilkens H Jany B Chronic thromboembolic pulmonary hypertension: Time delay from onset of symtoms to diagnosis and clinical condition at diagnosis Dtsch Med Wochenschr 2014 139 in press 10.1055/s-0034-1370256 25093951

[B10] Lewczuk J Piszko P Jagas J Porada A Wojciak S Sobkowicz B Wrabec K Prognostic factors in medically treated patients with chronic pulmonary embolism Chest 2001 119 818 823 10.1378/chest.119.3.818 11243963

[B11] Riedel M Stanek V Widimsky J Prerovsky I Longterm follow-up of patients with pulmonary thromboembolism: late prognosis and evolution of hemodynamic and respiratory data Chest 1982 81 151 158 10.1378/chest.81.2.151 7056079

[B12] Alhadad A Miniati M Alhadad H Gottsäter A Bajc M The value of tomographic ventilation/perfusion scintigraphy (V/PSPECT) for follow-up and prediction of recurrence in pulmonary embolism Thromb Res 2012 130 6 877 881 10.1016/j.thromres.2012.09.002 23026380

[B13] Oger E Incidence of venous thromboembolism: a community-based study in Western France. EPI-GETBP Study Group. Groupe d’Etude de la Thrombose de Bretagne Occidenatle Thromb Haemost 2000 83 5 657 660 10823257

[B14] De Monaco NA Dang Q Kapoor WN Ragni MW Pulmonary embolism incidence is increasing with use of spiral computed tomography Am J Med 2008 121 7 611 617 10.1016/j.amjmed.2008.02.035 18589057 PMC2711635

[B15] Tsai AW Cushman M Rosamond WD Heckbert SR Polak JF Folsom AR Cardiovascular risk factors and venous thromboembolism incidence: the Longitudinal Investigation of Thromboembolism Etiology Arch Intern Med 2002 162 1182 1189 10.1001/archinte.162.10.1182 12020191

[B16] Olschewski H Hoeper MM Borst MM Ewert R Grünig E Kleber F-X Kopp B Opitz C Reichenberger F Schmeisser A Schranz D Schulze-Neick I Wilkens H Winkler J Worth H Diagnostik und Therapie der chronischen pulmonalen Hypertonie Pneumologie 2006 60 749 771 10.1055/s-2006-954981 17163316

[B17] Held M Grün M Kaiser R Wilkens H Jany BH Cardiopulmonary exercise testing to detect Pulmonary Hypertension In Patients with Suspected Chronic Thromboembolic Pulmonary Hypertension And Normal Echocardiography Respiration 2014 87 379 387 doi:10.1159/000358565 24732343 10.1159/000358565

[B18] Coghlan GJG Denton CP Grünig E Bonderman D Distler O Khanna D Müller-Ladner U Pope JE Vonk MC Doelberg M Chafha-Boreham H Heinzl H Rosenberg DM Mc Laughlin VV Seibold JR DETECT study group Evidence-based detection of pulmonary arterial hypertension in systemic sclerosis. The DETECT study Ann Rheum Dis 2013 doi:10.1136/annrheumdis-2013-203301 10.1136/annrheumdis-2013-203301 PMC4078756 23687283

[B19] Grünig E Barner A Bell M Claussen M Dandel M Dumitrescu D Gorenflo M Holt S Kovacs G Ley S Meyer JF Pabst S Riemekasten G Saur J Schwaiblmair M Seck C Sinn L Sorichter S Winkler J Leuchte HH Non-invasive diagnosis of pulmonary hypertension: ESC/ERS Guidelines with updated Commentary of the Cologne Consensus Conference 2011 Int J Cardiol 2011 152 Supp. 1 S 3 S12 10.1016/S0167-5273(11)70488-0 22221971

[B20] Fisher MR Forfia PR Chamera E Housten-Harris T Champion HC Girgis RE Corretti MC Hassoun PM Accuracy of Doppler echocardiography in the haemodynamic assessment of pulmonary hypertension Am J Respir Crit Care Med 2009 179 615 621 10.1164/rccm.200811-1691OC 19164700 PMC2720125

[B21] Hinderliter AL Willis PW Barst RJ Rich S Rubin LJ Badesch DB Groves BM McGoon MD Tapson VF Bourge RC Brundage BH Koerner SK Langleben D Keller CA Murali S Uretsky BF Koch G Li S Clayton LM Jöbsis MM Blackburn SD Jr Crow JW Long WA Primary pulmonary hypertension Study Group. Effects of long-term infusion of prostacyclin (epoprostenol) on echocardiographic measures of right ventricular structure and function in primary pulmonary hypertension Circulation 1997 95 1479 1486 10.1161/01.CIR.95.6.1479 9118516

[B22] Fisher MR Criner GJ Fishman AP Assoun PM Minai OA Scharf SM Fessler HE NETT Research Group. Estimating pulmonary artery pressures by echocardiography in patients with emphysema Eur Respir J 2007 30 914 921 10.1183/09031936.00033007 17652313

[B23] Scheidl SJ Englisch C Kovacs G Reichenberger F Schulz R Breithecker A Ghofrani H-A Seeger W Olschewski H Diagnosis of CTEPH versus iPAH using capillary to end-tidal carbon dioxide gradients Eur Respir J 2012 39 119 124 10.1183/09031936.00109710 21737552

[B24] Ryan KL Fedullo PF Davies GB Vazquez TE Moser KM Perfusion scan findings understate the severity of angiographic and hemodyanmic compromise in chronic thromboembolic pulmonary hypertension Chest 1988 93 1180 1185 10.1378/chest.93.6.1180 3371097

[B25] Azarian R Wartski M Collignon MA Parent F Hervé P Sors H Simonneau G Lung perusion scans and haemodynamics in acute and chronic pulmonary embolism J Nucl Med 1997 38 980 983 9189155

[B26] Galiè N Hoeper MM Humbert M Torbicki A Vachiery JL Barbera JA Beghetti M Corris P Gaine S Gibbs JS Gomez-Sanchez MA Jondeau G Klepetko W Opitz C Peacock A Rubin L Zellweger M Simonneau G ESC Commitee for Practice Guidelines (CPG) Guidelines for the diagnosis and treatment of pulmonary hypertension: the Task Force for the Diagnosis and Treatment of Pulmonary Hypertension of the European Society of Cardiology (ESC) and the European Respiratory Society (ERS), endorsed by the International Society of Heart and Lung Transplantation (ISHLT) Eur Heart J 2009 30 2493 2537 19713419 10.1093/eurheartj/ehp297

[B27] Wilkens H Lang I Behr J Grohe C Guth S Hoeper MM Kramm T Krüger U Langer F Rosenkranz S Schäfers HJ Schmidt M Seyfarth HJ Wahlers T Worth H Mayer E Chronic thromboembolic pulmonary hypertension (CTEPH): Updated recommendation of the Cologne Consensus Conference 2011 Int J Cardiol 2011 152 Supp. 1 S 54 S 60 10.1016/S0167-5273(11)70493-4 22221974

[B28] Rudski LG Lai WW Afilalo J Hua L Handschumacher MD Chandrasekaran K Solomon SD Louie EK Schiller NB Guidelines for the echocardiographic assessment of the right heart in adults: a report from the American Society of Echocardiography. Endorsed by the European Association of Echocardiography, a registered branch of the European Society of Cardiology, and the Canadian Society of Echocardiography J Am Soc Echocardiogr 2010 23 685 713 10.1016/j.echo.2010.05.010 20620859

[B29] American Thoracic Society/American College of Chest Physicians ATS/ACCP-Statement on cardiopulmonary exercise testing Am J Respir Crit Care Med 2003 167 211 277 12524257 10.1164/rccm.167.2.211

[B30] Meyer FJ Borst MM Buschmann HC Ewert R Friedmann-Bette B Ochmann U Petermann W Preissler AM Rohde D Rühler KH Sorichter S Stähler G Westhoff M Worth H Belastungsuntersuchungen in der Pneumologie. Empfehlungen der Deutschen Gesellschaft für Pneumologie und Beatmungsmedizin e. V. Exercise Testing in Respiratory Medicine. DGP Recommendations Pneumologie 2013 67 16 3423325729

[B31] Rosenkranz S Behr J Ewert R Ghofrani HA Grünig E Halank M Hoeper MM Leuchte HH Olschewski H Schmeisser A Speich R Wilkens H Opitz CF Rechtsherzkatheter-Untersuchung bei pulmonaler Hypertonie Dtsch Med Wochenschr 2011 136 2601 2620 22160954 10.1055/s-0031-1292858

[B32] Moser KM Auger WR Fedullo PF Chronic major vessel thromboembolic pulmonary hypertension Circulation 1990 81 1735 1743 10.1161/01.CIR.81.6.1735 2188751

