# Supplementary material for: A symptom-related monitoring program following pulmonary embolism for the early detection of CTEPH: a prospective observational registry study
Source: BMC Pulm Med. 2014 Aug 28;14:141. doi: 10.1186/1471-2466-14-141 (PMC4152276; doi:10.1186/1471-2466-14-141)
Supplement: Additional file 1 — 5 Item questionnaire, original German version. [file 1471-2466-14-141-S1.pdf]

Telefonvisite 1 Zeitpunkt 3 Monate nach LE
Datum

Patientenaufkleber	
Laufende Nummer	
Ident-Nummer	

Besteht Luftnot unter Ruhebedingungen ?

Ja □ Nein □

Besteht Luftnot unter Belastungsbedingungen ?

Ja □ Nein □

Besteht ein Druckgefühl im Brustkorb ?

Ja □ Nein □

Abgeschätzes WHO-Funktionsstadium?

WHO I II III IV

Besteht Schindel?

Ja □ Nein □

- **Wenn ja?** □
- **Drehschwindel ?** □
- **Schwankschwindel ?** □
- **„Schwarzwerden vor den Augen“?** □

Kam es seit der Krankenhausentlassung zu einer Bewusstlosigkeit?

Ja □ Nein □

Einbestellung nötig?

Ja □ Nein □ Einbestelldatum □

Bemerkung
