# Supplementary material for: A symptom-related monitoring program following pulmonary embolism for the early detection of CTEPH: a prospective observational registry study
Source: BMC Pulm Med. 2014 Aug 28;14:141. doi: 10.1186/1471-2466-14-141 (PMC4152276; doi:10.1186/1471-2466-14-141)
Supplement: Additional file 2 — 5 Item questionnaire, translated English version. [file 1471-2466-14-141-S2.pdf]

5 Item Questionnaire

Questions used at the tree and six months follow-up:

1. Do you experience shortness of breath at rest ?
2. Do you feel shortness of breath while exercising ?
3. Do you have chest pain ?
4. Do you feel dizzy ?
5. Did you faint or lose consciousness during the last three months?

Answer is Yes/No

Questions used at the twelve months follow-up:

1. Do you experience shortness of breath at rest ?
2. Do you feel shortness of breath while exercising?
3. Do you have chest pain?
4. Do you feel dizzy?
5. Did you faint or lose consciousness during the last six months?

Answer is Yes/No
